# Protein Docking by the Interface Structure Similarity: How Much Structure Is Needed?

**DOI:** 10.1371/journal.pone.0031349

**Published:** 2012-02-13

**Authors:** Rohita Sinha, Petras J. Kundrotas, Ilya A. Vakser

**Affiliations:** 1 Center for Bioinformatics, The University of Kansas, Lawrence, Kansas, United States of America; 2 Department of Molecular Biosciences, The University of Kansas, Lawrence, Kansas, United States of America; Koç University, Turkey

## Abstract

The increasing availability of co-crystallized protein-protein complexes provides an opportunity to use template-based modeling for protein-protein docking. Structure alignment techniques are useful in detection of remote target-template similarities. The size of the structure involved in the alignment is important for the success in modeling. This paper describes a systematic large-scale study to find the optimal definition/size of the interfaces for the structure alignment-based docking applications. The results showed that structural areas corresponding to the cutoff values <12 Å across the interface inadequately represent structural details of the interfaces. With the increase of the cutoff beyond 12 Å, the success rate for the benchmark set of 99 protein complexes, did not increase significantly for higher accuracy models, and decreased for lower-accuracy models. The 12 Å cutoff was optimal in our interface alignment-based docking, and a likely best choice for the large-scale (e.g., on the scale of the entire genome) applications to protein interaction networks. The results provide guidelines for the docking approaches, including high-throughput applications to modeled structures.

## Introduction

Computational methods for structural modeling of protein-protein interactions (PPI) are important as a source of structural information on protein complexes that complements experimentally determined structures, and as a way to understand the mechanisms of protein association. The computational approaches to prediction of the structures of protein-protein complexes (protein docking) rely on the experimentally derived characterization of protein-protein interfaces, such as high degree of steric complementarity, physicochemical properties, residue propensities, etc [1]. However, traditionally, they have not directly utilized experimentally determined structures as modeling templates [2]. The increasing availability of the co-crystallized protein-protein complexes makes such template-based modeling/docking possible [3]–[9].

The template-based docking is complementary to the free docking [6]. Its relative value will naturally grow with more protein-protein templates/complexes determined experimentally. Protein-protein template-based approaches based on sequence similarity currently can account for ∼20% of known PPI [10], [11]. Threading techniques provide another valuable tool for PPI modeling [5]. At the same time, structure alignment techniques are important for the detection of remote target-template similarities [12]. Such an alignment may be performed between the whole target and template structures, or between the whole target and the templates interfaces. The latter approach assumes that the structural similarity may be more easily detected at the binding site, rather than for the whole protein (where it may not exist at all). Both approaches have their advantages, based on the observed relationships between local vs. global similarities in interacting proteins, which is the subject of our current studies (Kundrotas et al., in preparation).

Methodology described in this report is based on the structure alignment of the interfaces. A number of studies focus on distinct geometric and physicochemical properties of protein-protein interfaces [13]–[17]. Methods for binding site identification and comparison [18] are based on search for the surface clefts [17], [19], surface matching algorithms [6], [20]–[22] and structure and/or sequence patterns [19], [23]–[30]. Structural interface conservation was used to predict PPI [31]–[33], binding sites [33]–[35], and druggable hot spots [36]. An important development in this field is the procedures and analysis tools explicitly related to modeled structures of limited accuracy [19], [24], [37]–[40].

The success of the approach by definition hinges on the way the interface is defined in terms of its structural content. A number of definitions of the interfaces are most often based on the change in solvent accessible surface area upon binding or on various types of distance cutoffs across the interface. Varying definitions significantly influence the size and the composition of the interfaces, thus having a major effect on the interface alignment. This paper provides in-depth account of a systematic study (briefly mentioned in our short advance report [6]) to find the optimal definition/size of the interfaces for the structure alignment-based docking applications.

## Results and Discussion

### Libraries of interface fragments

Defining interfaces for structural alignment based on the residues in direct physical contact only may lead to wrong results due to the loss of significant structural details at the interface. On the other hand, large distance cutoffs may impair ability to find local structural similarity at the interface due to the presence of large non-interface parts (in the extreme case, the entire protein structure). Thus, selection of the cutoff distance for the interface definition in the context of the structural alignment can be considered as optimization.

**Figure 1:**
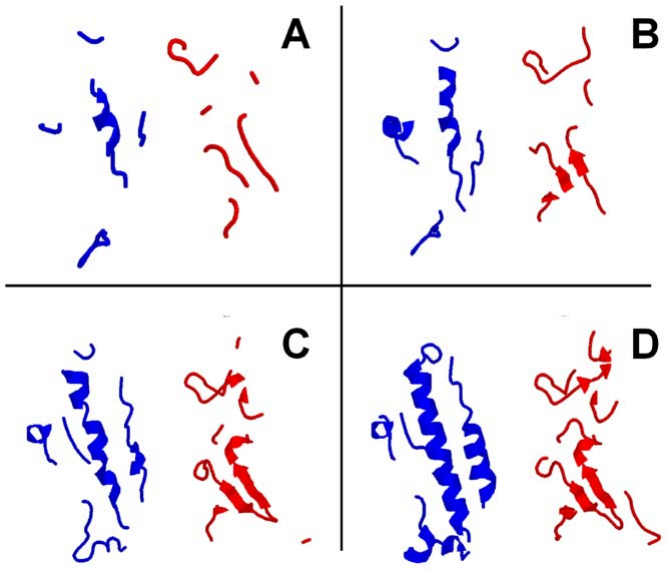
Example of interface fragments corresponding to different cutoff values. Fragments of 1bp3 complex were extracted using interface cutoffs: (A) 6 Å, (B) 8 Å, (C) 10 Å, and (D) 12 Å. Ligand (the smaller protein in the complex) is in blue and Receptor (the larger protein in the complex) is in red.

In this study, we adopted the interface definition based on the distance between any atoms across the interface. To find the optimal distance, we generated five interface libraries with different values of the distance: 6 Å, 8 Å, 10 Å, 12 Å and 16 Å (see Methods). Figure 1 shows an example of interface fragments in 1bp3 complex corresponding to different cutoff distances. One can clearly see the gradual appearance of the secondary structure elements as the cutoff value increases. The interface of the first protein in the complex (blue ribbons in Figure 1) largely consists of two α-helixes (residues G161–S184 and H18–Y28) interacting with β-sheet (β-strands W272-V279 and D291–V297) and loop fragments (residues Y240–M248, K385-W391, L202–I209 and P329–E366) from the second protein (red ribbons in Figure 1). However, the fragment from the 6 Å library (Figure 1A) contains only a short fragment (residues D171–I179) of one of the α-helixes and the β-sheet structure of the second component is indiscernible with only short fragments (S270-T274 and E292-Y294) visible. Such representation is clearly inadequate for the successful structural alignment that involves secondary structure elements. The fragment from the 8 Å library (Figure 1B) has longer α-helix (D171-R183) in the first protein and visible β-sheet-like structure in the second component, but the second α-helix of the first protein still remains obscure. The fragment from the 10 Å library (Figure 1C) already shows one full α-helix in the first protein and the complete β-sheet structure in the second protein. Yet, the second α-helix from the first protein (residues Q22-D26) is only partially visible. Only the fragment from the 12 Å library reveals the complete structural details of the interface (Figure 1D). Further increase of the distance leads to inclusion of significant non-interface parts of protein structure (the effect already seen in Figure 1C and 1D). Similar trend was observed in other interface library entries.

### Structural alignment with interfaces

The modeling procedure aligns separate structures of unbound target proteins (‘receptor’ and ‘ligand’ defined as the larger and the smaller proteins in the complex) with the library of co-crystallized interfaces. The C^α^-only alignment was performed by TM-align [41] (see Methods). The C^α^ alignment reduces the effect of conformational changes upon binding, thus enabling proper overlap of the unbound and bound fragments.

Structural deficiencies in the fragments from smaller cutoff libraries are reflected in the lower TM-scores [41], [42] (see Methods) for the alignments between such fragments and the target structures, thus substantially reducing the rank of the correct models. For example, 1bp3 complex (interface shown in Figure 1) is structurally homologous to a target complex 3 hhr (TM-scores 0.8 and 0.7 for structural alignments of entire 1bp3 and 3 hhr receptors and ligands, respectively, with corresponding sequence identities 31% and 66%). However, the 1bp3 interface fragment from the 6 Å library did not generate any models for the 3 hhr target due to TM-scores that were below statistical significance threshold (0.15 and 0.2 for the receptors and ligands, correspondingly). On the other hand, models generated using 1bp3 fragments from the 8 Å, 10 Å, 12 Å and 16 Å libraries had root mean square deviation between ligand interface C^α^ atoms in the model and in the native complex (*i*-RMSD) 4.18 Å, 4.22 Å, 4.22 Å and 4.3 Å correspondingly. However, the 8 Å library model was ranked 42 among all 8 Å library models generated for this target, whereas model ranked 1 had *i*-RMSD = 38.0 Å. Only models built using interface libraries with adequate structural details (10 Å, 12 Å and 16 Å libraries) were ranked 1 by the TM-score. Interestingly, similar trend holds even for highly similar proteins. For example, 1eay template complex is very similar to the target complex 1a0o (TM-scores 0.8 and 0.9 for structural alignments of the entire 1a0o and 1eay receptors and ligands, respectively, with corresponding sequence identities 96% and 100%). However, 1eay interface fragment from the 6 Å library could not generate statistically significant alignments for the 1a0o target (TM-scores 0.35 and 0.07). Models generated using the 1eay fragments from 8 Å, 10 Å, 12 Å and 16 Å libraries had *i*-RMSD = 1.5 Å, 1.7 Å, 2.0 Å and 2.2 Å, respectively. However, 8 Å and 10 Å libraries models were ranked 818 and 35 respectively, whereas the 12 Å and 16 Å library models were ranked 5 and 1. Thus, 12 Å and 16 Å libraries provided correct models for the 1a0o target within top 10 predictions. The *i*-RMSD values for the 12 Å and 16 Å libraries models were similar to RMSD between the entire structures of bound 1eay and unbound 1a0o complexes (2.2 Å).

Relatively poor ranking of models from the small cutoff libraries was because the small fragments lacking well-defined secondary structure elements can be aligned to a random place in the target structure (thus generating models with high TM-score but large *i*-RMSD). At the same time, alignment of such fragment of a bound protein to the unbound target interface may have significantly lower TM-score. This is especially true if there is a significant conformational change between bound and unbound structures. As shown in Figure 1, the distance of 12 Å and above provides full structural details of the interfaces. Thus, it reduces the possibility of the “good” random alignment and enhances the TM-score of the correct alignment by increasing parts of well-aligned interface areas.

### Modeling success rates for different interface libraries

To validate the docking, we used the Dockground benchmark set, for which both monomers have both bound and unbound structures available [43]. The quality of the resulting models was accessed by root mean square deviation between ligand interface C^α^ atoms in the model and in the native complex (*i*-RMSD), based on the optimal alignment of the receptor structures (see Methods for details).

**Figure 2:**
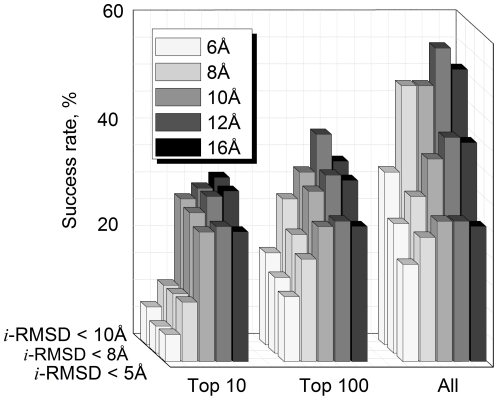
Docking success rates for different interface libraries. The docking was performed on the Dockground benchmark set. The success rate is defined as percentage of target complexes for which at least one match is in top 10, top 100, and in all matches generated for the target has *i*-RMSD≤5, 8, and 10 Å. The results are shown for 6, 8, 10, 12, and 16 Å interface libraries (see the text for details).

The models were generated and evaluated using our five interface libraries. Results presented in Figure 2 are the success rates defined as percentage of target complexes for which at least one model within a certain pool (top 10, top 100, and all models generated for the target) has *i*-RMSD≤5, 8, and 10 Å. The *i*-RMSD≤5 Å is comparable with the criteria for discriminating acceptable-quality models of protein-protein complexes in CAPRI [44]. Analysis of the docking funnels [45] suggests that the models with *i*-RMSD up to 8–10 Å can be locally minimized/refined to the near native structures.

The data in Figure 2 shows that the success rates for the 10 Å, 12 Å and 16 Å libraries are significantly higher than those for the 6 Å and 8 Å libraries (see discussion above). The 12 Å library models consistently had high success rates. In the case of relaxed acceptance criteria for 16 Å library docking, the matches with *i*-RMSD≤10 Å were in top 10 predictions, whereas models from the 12 Å library had rank significantly worse than 10. This was the case for 1he8 docking using 16 Å (model ranked 4 with *i*-RMSD 6.3 Å) and 12 Å (model ranked 19 with *i*-RMSD 6.0 Å) template fragments from 1k8r, and for 2g45 docking using 16 Å templates fragments from 1nbf (model ranked 4 with *i*-RMSD 9.5 Å) and 12 Å template fragments from 1tgz (model ranked 74 with *i*-RMSD 9.7 Å).

**Figure 3:**
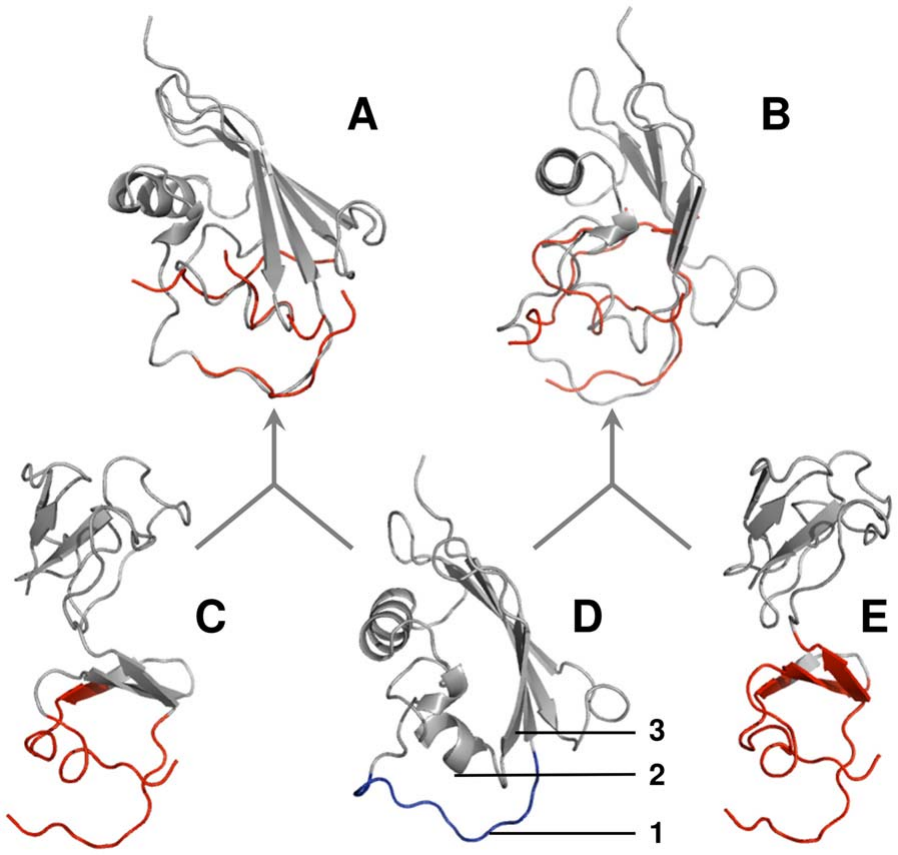
Example of docking based on 12 Å and 16 Å interface libraries. 3sic ligand (gray ribbons in A, B, D) was aligned with fragments of 1oyv ligand (red) extracted using 12 Å (A) and 16 Å (B) interface cutoffs. For comparison, the entire structure of 1oyv ligand is shown with 12 Å (C) and 16 Å (E) fragments (red). The entire structure of 3sic ligand with the loop participating in binding (blue) is shown in D. Binding loop in 3sic ligand is marked 1, and α-helix and β-sheet closest to this loop are marked 2 and 3, respectively.

For some targets, the 16 Å library was unable to generate an acceptable model while the 12 Å library (smaller fragments) succeeded. An example of such case is shown in Figure 3 where models for the ligand in 3sic were generated using ligand fragments from 1oyv. As the figure shows, the structures of 3sic and 1oyv ligands have dissimilar folds (TM-score for the alignment of the entire ligand structures is 0.7 with overall sequence identity 66%). The 3sic ligand is trypsin inhibitor with the “classic” binding loop (residues E67-D76, marked 1 in Figure 3D). The secondary structure elements closest to this loop are α-helix and β-sheet (marked 2 and 3 in Figure 3D). The 12 Å library fragment from the 1ovy ligand (red ribbons in Figure 3C) contain an α-helix-like loop (residues T88-G93), which aligns well with the α-helix in the 3sic ligand (Figure 3A). The orientation of two other binding loops in the 1oyv ligand relative to this α-helix-like loop is similar to the relative orientations of the binding loop and α-helix in the 3sic ligand, yielding an accurate model for the 3sic target (*i*-RMSD 1.1 Å with rank 3). The 1oyv fragment from the 16 Å library (red ribbons in Figure 3E) contains a significant part of non-interface β-sheet, which aligns with the β-sheet in the 3sic ligand (Figure 3B). Since orientations of these β-sheets relative to the binding site are different for the 3sic and 1oyv ligands, the resulting model has significantly larger *i*-RMSD = 7.0 Å. The model was not acceptable because more than 50% of the structural alignment contains non-surface residues of the target protein (this criterion is required to insure that the interface fragments do not align with the core of proteins producing random output, see above).

Increase of the distance cutoff defining the interface leads eventually to inclusion of the entire monomer structures, thus transforming partial structural alignment into full structure alignment. The detailed comparison of the partial (interface only) and the full protein structure alignment is a subject of a separate study (Kundrotas et al., in preparation). In the context of this report we would like to mention that the overall success rates there follow essentially the same trend as shown in Figure 2 for the 12 Å and 16 Å libraries, i.e. tend to decrease for the full-structure alignment models, especially with relaxed model acceptance criteria (larger *i*-RMSD and less demanding top ranking). Generally, the partial and the full structural alignments are applicable to different types of target/template similarity.

General utility of the docking approaches requires applicability to experimentally determined as well as modeled structures of monomers of limited accuracy, especially in large-scale (e.g., genome-wide) modeling of protein networks. Such approaches have to be fast (high-throughput) and tolerant to significant structural inaccuracies of the monomers [46]. Overall, the 12 Å cutoff appears to be optimal for the relaxed model acceptance criteria needed for docking of modeled structures. It also provides faster alignment than the one with larger cutoffs. Thus, it is well suited for the high-throughput structural modeling of protein-protein complexes in large PPI networks.

Overall, the structure-based alignment docking has a higher success rate on the unbound benchmark sets than the free docking [6]. Its utility will further grow with increasing availability of the experimentally determined templates.

### Conclusions

A large-scale systematic benchmarking of docking methodology based on the structural alignment of protein interfaces was performed to determine the optimal size of the structure in the alignment. The results showed that structural areas corresponding to the cutoff values ≤10 Å across the interface inadequately represented structural details of the interfaces. The use of such areas in the modeling significantly reduced docking success rates. With the increase of the cutoff beyond 12 Å, the success rate for our dataset of 99 protein complexes did not increase significantly for higher accuracy models, and decreased for lower-accuracy models. While larger structural segments (full structures at the extreme) could provide better alignment for some complexes, the modeling time for aligning larger fragments increases. The 12 Å cutoff was optimal in our interface alignment-based docking, and a likely best choice for the large-scale (e.g., on the scale of the entire genome) applications to protein interaction networks. Such systems contain only a limited number of experimentally determined monomer structures and by necessity are populated by monomer models of limited accuracy obtained by high-throughput computational techniques. Thus, they require relaxed docking acceptance criteria where the 12 Å cutoff provides the best results.

## Methods

The interface definition was based on the distance between any atoms across the interface. The interfaces were obtained from the set of pairwise complexes generated by the Dockground resource (http://dockground.bioinformatics.ku.edu) [43], [47] with the following parameters. The X-ray resolution of the structures had to be <3 Å, they had to come from at least dimeric biological unit, and the sequence identity between different complexes had to be <90%. The selection resulted in 11,932 complexes. The interface backbone atoms were extracted and stored in libraries of interfaces. An interface residue was defined as the one having at least one atom within a certain distance (varied from 6 to 16 Å) of any atom of the other protein in the complex.

The C^α^-only structural alignment of the target proteins with the co-crystallized interfaces was performed by TM-align [41]. TM-align was chosen over many other available structural alignment programs mainly due to its superior ability to align remotely related proteins (including structural fragments with non-continuous sequences) and its speed that makes it suitable for large-scale calculations. For comparison, we also carried out structure alignment for several targets by another popular program SKA [48] and found no essential differences in the resulting models. The quality of the alignment was assessed by TM-score [42], which has values in 0 to 1 range. We modified the original TM-align code for aligning discontinuous fragments of polypeptide chains, to limit the summation to the template interface and the corresponding aligned target residues in the TM-score calculation. This makes it equivalent to the *i*TM-score, recently introduced by Gao and Skolnick [49]. In general, TM-scores <0.2 indicate no fold similarity, whereas scores >0.5 point to similar folds [41], [42]. Significant alignments were defined according to the criteria: (*i*) TM-score of at least one alignment >0.4, (*ii*) at least 50% of aligned residues for both receptor and ligand should be on the protein surface, and (*iii*) at least 40% of residues in both interface components should be included in the alignments. Transformation matrices from each significant alignment were applied to the target receptor and ligand to generate the models. The docking protocol for two proteins, involving search through the entire library of interfaces and generation of a full set of matches (the number varies according to the availability of templates), takes several hours on a single core processor.

The pre-generated Dockground benchmark set [43] containing 99 protein-protein complexes (27 enzyme-inhibitor, 6 antibody-antigen, 2 cytokine or hormone/receptors, and 64 other complexes), for which both monomers have both bound and unbound structures available, was used for validation of the docking results. Comparison of template-based and template-free docking, based on this benchmark set, was reported earlier [6]. Target self-hits were excluded from consideration. Only non-homologous templates were identified for 26 targets (hard cases for homology modeling). Quality of the resulting models was accessed by *i*-RMSD between ligand interface C^α^ atoms in the model and in the native complex after the optimal alignment of the receptors. The distance threshold for the interface residues in the *i*-RMSD calculations was 6 Å. Sequence identities between target and template were calculated by CLUSTALW [50]. Rank of a model was based on the sum of the alignment scores (TM-score) for the target monomers and the template interfaces.

## References

[1] Vakser IA, Kundrotas P (2008). Predicting 3D structures of protein-protein complexes.. Curr Pharm Biotech.

[2] Janin J, Henrick K, Moult J, Ten Eyck L, Sternberg MJE (2003). CAPRI: A Critical Assessment of PRedicted Interactions.. Proteins.

[3] Russell RB, Alber F, Aloy P, Davis FP, Korkin D (2004). A structural perspective on protein–protein interactions.. Curr Opin Struct Biol.

[4] Gunther S, May P, Hoppe A, Frommel C, Preissner R (2007). Docking without docking: ISEARCH - prediction of interactions using known interfaces.. Proteins.

[5] Lu L, Lu H, Skolnick J (2002). MULTIPROSPECTOR: An algorithm for the prediction of protein-protein interactions by multimeric threading.. Proteins.

[6] Sinha R, Kundrotas PJ, Vakser IA (2010). Docking by structural similarity at protein-protein interfaces.. Proteins.

[7] Korkin D, Davis FP, Alber F, Luong T, Shen M (2006). Structural modeling of protein interactions by analogy: Application to PSD-95.. PLoS Comp Biol.

[8] Zacharias M (2010). Accounting for conformational changes during protein–protein docking.. Curr Opin, Struct Biol.

[9] Ogmen U, Keskin O, Aytuna AS, Nussinov R, Gursoy A (2005). PRISM: protein interactions by structural matching.. Nucleic Acids Research.

[10] Kundrotas PJ, Alexov E (2006). Predicting 3D structures of transient protein-protein complexes by homology.. Bioch Biophys Acta.

[11] Kundrotas PJ, Lensink MF, Alexov E (2008). Homology-based modeling of 3D structures of protein-protein complexes using alignments of modified sequence profiles.. Int J Biol Macromol.

[12] Hasegawa H, Holm L (2009). Advances and pitfalls of protein structural alignment.. Curr Opin, Struct Biol.

[13] Reichmann D, Rahat O, Cohen M, Neuvirth H, Schreiber G (2007). The molecular architecture of protein–protein binding sites.. Curr Opin Struct Biol.

[14] Jones S, Thornton JM (1997). Analysis of protein-protein interaction sites using surface patches.. J Mol Biol.

[15] Tuncbag N, Gursoy A, Guney E, Nussinov R, Keskin O (2008). Architectures and functional coverage of protein–protein interfaces.. J Mol Biol.

[16] Chakrabarti P, Janin J (2002). Dissecting protein-protein recognition sites.. Proteins.

[17] Nicola G, Vakser IA (2007). A simple shape characteristic of protein-protein recognition.. Bioinformatics.

[18] Zhou HX, Qin S (2007). Interaction-site prediction for protein complexes: A critical assessment.. Bioinformatics.

[19] Binkowski TA, Joachimiak A, Liang J (2005). Protein surface analysis for function annotation in high-throughput structural genomics pipeline.. Protein Sci.

[20] Keskin O, Nussinov R, Gursoy A (2008). PRISM: Protein-protein interaction prediction by structural matching.. Methods Mol Biol.

[21] La D, Esquivel-Rodriguez J, Venkatraman V, Li B, Sael L (2009). 3D-SURFER: Software for high-throughput protein surface comparison and analysis.. Bioinformatics.

[22] Konc J, Janezic D (2010). ProBiS algorithm for detection of structurally similar protein binding sites by local structural alignment.. Bioinformatics.

[23] Fetrow JS, Siew N, Di Gennaro JA, Martinez-Yamout M, Dyson HJ (2001). Genomic-scale comparison of sequence- and structure-based methods of function prediction: Does structure provide additional insight?. Protein Sci.

[24] Stark A, Shkumatov A, Russell RB (2004). Finding functional sites in structural genomics proteins.. Structure.

[25] Pazos F, Sternberg MJE (2004). Automated prediction of protein function and detection of functional sites from structure.. Proc Natl Acad Sci USA.

[26] Ofran Y, Rost B (2003). Predicted protein-protein interaction sites from local sequence information.. FEBS Lett.

[27] Wilkins AD, Lua R, Erdin S, Ward RM, Lichtarge O (2010). Sequence and structure continuity of evolutionary importance improves protein functional site discovery and annotation.. Protein Sci.

[28] Glaser F, Pupko T, Paz I, Bell RE, Bechor-Shental D (2003). ConSurf: Identification of functional regions in proteins by surface-mapping of phylogenetic information.. Bioinformatics.

[29] Rossi A, Marti-Renom MA, Sali A (2006). Localization of binding sites in protein structures by optimization of a composite scoring function.. Protein Sci.

[30] Fernandez-Recio J, Totrov M, Skorodumov C, Abagyan R (2005). Optimal docking area: A new method for predicting protein-protein interaction sites.. Proteins.

[31] Aytuna AS, Gursoy A, Keskin O (2005). Prediction of protein-protein interactions by combining structure and sequence conservation in protein interfaces.. Bioinformatics.

[32] Gursoy A, Tuncbag N, Keskin O (2011). Prediction of protein-protein interactions: unifying evolution and structure at protein interfaces.. Physical Biology.

[33] Zhang QC, Petrey D, Norel R, Honig BH (2010). Protein interface conservation across structure space.. Proc Natl Acad Sci U S A.

[34] Keskin O, Nussinov R (2007). Similar binding sites and different partners: Implications to shared proteins in cellular pathways.. Structure.

[35] Mitchell EM, Artymiuk PJ, Rice DW, Willett P (1990). Use of Techniques Derived from Graph-Theory to Compare Secondary Structure Motifs in Proteins.. Journal of Molecular Biology.

[36] Kozakov D, Hall DR, Chuang GY, Cencic R, Brenke R (2011). Structural conservation of druggable hot spots in protein-protein interfaces.. Proceedings of the National Academy of Sciences of the United States of America.

[37] Arakaki AK, Zhang Y, Skolnick J (2004). Large-scale assessment of the utility of low-resolution protein structures for biochemical function assignment.. Bioinformatics.

[38] Kundrotas PJ, Vakser IA (2010). Accuracy of protein-protein binding sites in high-throughput template-based modeling.. PLoS Comp Biol.

[39] Tovchigrechko A, Wells CA, Vakser IA (2002). Docking of protein models.. Protein Sci.

[40] Cammer SA, Hoffman BT, Speir JA, Canady MA, Nelson MR (2003). Structure-based active site profiles for genome analysis and functional family subclassification.. J Mol Biol.

[41] Zhang Y, Skolnick J (2005). TM-align: A protein structure alignment algorithm based on the TM-score.. Nucl Acid Res.

[42] Zhang Y, Skolnick J (2004). Scoring function for automated assessment of protein structure template quality.. Proteins.

[43] Gao Y, Douguet D, Tovchigrechko A, Vakser IA (2007). DOCKGROUND system of databases for protein recognition studies: Unbound structures for docking.. Proteins.

[44] Lensink MF, Wodak SJ (2010). Docking and scoring protein interactions: CAPRI 2009.. Proteins.

[45] Hunjan J, Tovchigrechko A, Gao Y, Vakser IA (2008). The size of the intermolecular energy funnel in protein-protein interactions.. Proteins.

[46] Sali A, Glaeser R, Earnest T, Baumeister W (2003). From words to literature in structural proteomics.. Nature.

[47] Douguet D, Chen HC, Tovchigrechko A, Vakser IA (2006). DOCKGROUND resource for studying protein-protein interfaces.. Bioinformatics.

[48] Petrey D, Xiang ZX, Tang CL, Xie L, Gimpelev M (2003). Using multiple structure alignments, fast model building, and energetic analysis in fold recognition and homology modeling.. Proteins.

[49] Gao M, Skolnick J (2011). New benchmark metrics for protein-protein docking methods.. Proteins-Structure Function and Bioinformatics.

[50] Larkin MA, Blackshields G, Brown NP, Chenna R, McGettigan PA (2007). Clustal W and Clustal X version 2.0.. Bioinformatics.

